# Supplementary material for: A single neuron in C. elegans orchestrates multiple motor outputs through parallel modes of transmission
Source: bioRxiv. 2023 Apr 2:2023.04.02.532814. Preprint. [Version 1] doi: 10.1101/2023.04.02.532814 (PMC10081309; doi:10.1101/2023.04.02.532814)
Supplement: 1 [file NIHPP2023.04.02.532814v1-supplement-1.pdf]

bioRxiv preprint doi: https://doi.org/10.1101/2023.04.02.532814; this version posted April 2, 2023. The copyright holder for this preprint (which was not certified by peer review) is the author/funder, who has granted bioRxiv a license to display the preprint in perpetuity. It is made available under aCC-BY 4.0 International license.

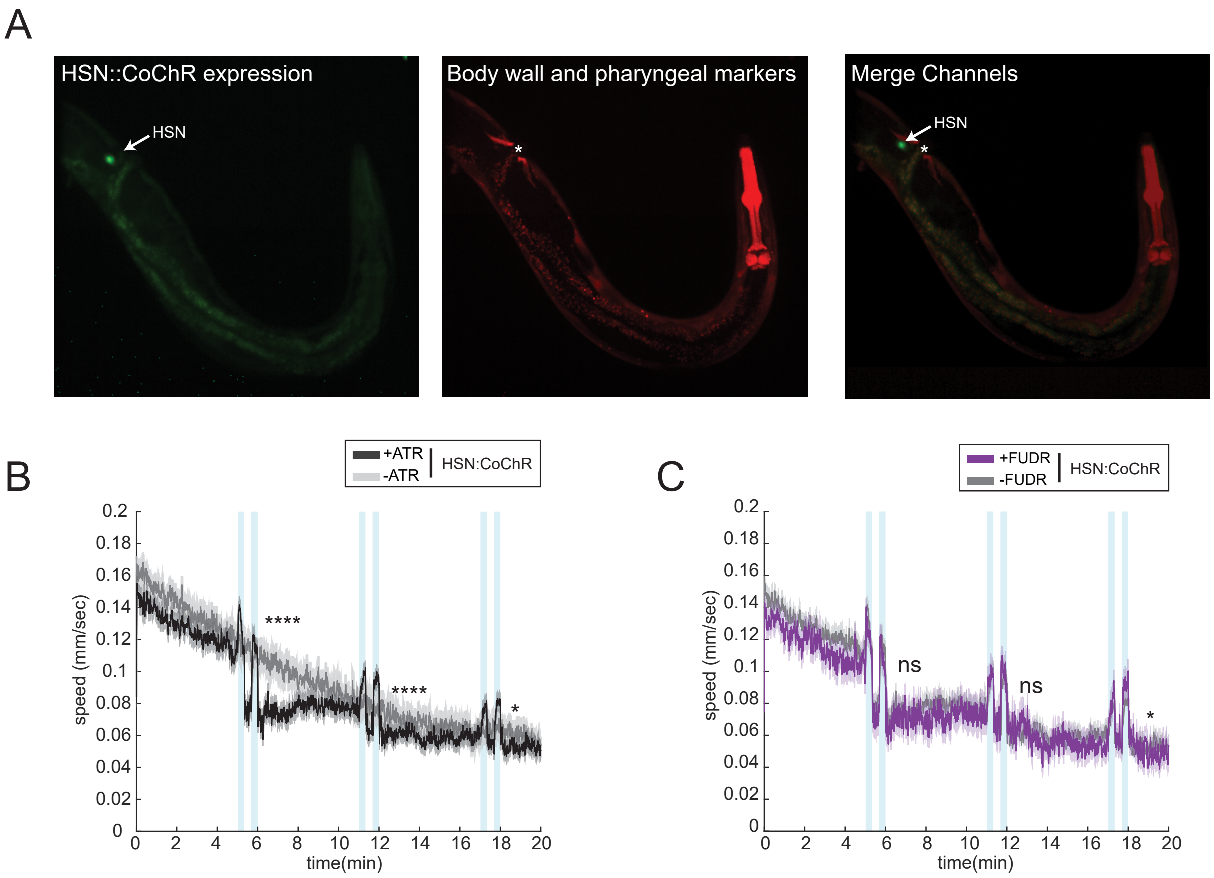


**Figure S1, Related to Figure 1**

(A) Representative images of HSN::CoChR-sl2-GFP expression (left), along with body wall and pharyngeal markers (middle), and a composite image (right). Arrows indicate HSN soma. Asterisks indicate vulva position.

(B) Full recordings of animal speed during several bouts of HSN::CoChR stimulation for animals recently transferred to plates with food. Data are shown for HSN::CoChR animals with and without ATR. . N= 195 animals for the ATR group, and 159 animals for the no-ATR control. *p<0.05, ****p<0.0001, Bonferroni-corrected t-test.

(C) Animal speed during several bouts of HSN::CoChR stimulation for animals recently transferred to food plates. Data are shown for HSN::CoChR animals either treated with FUDR or not. . N= 69 animals for the FUDR-treated group, and 195 animals for the no-FUDR control. *p<0.01, Bonferroni-corrected t-test.

bioRxiv preprint doi: https://doi.org/10.1101/2023.04.02.532814; this version posted April 2, 2023. The copyright holder for this preprint (which was not certified by peer review) is the author/funder, who has granted bioRxiv a license to display the preprint in perpetuity. It is made available under aCC-BY 4.0 International license.

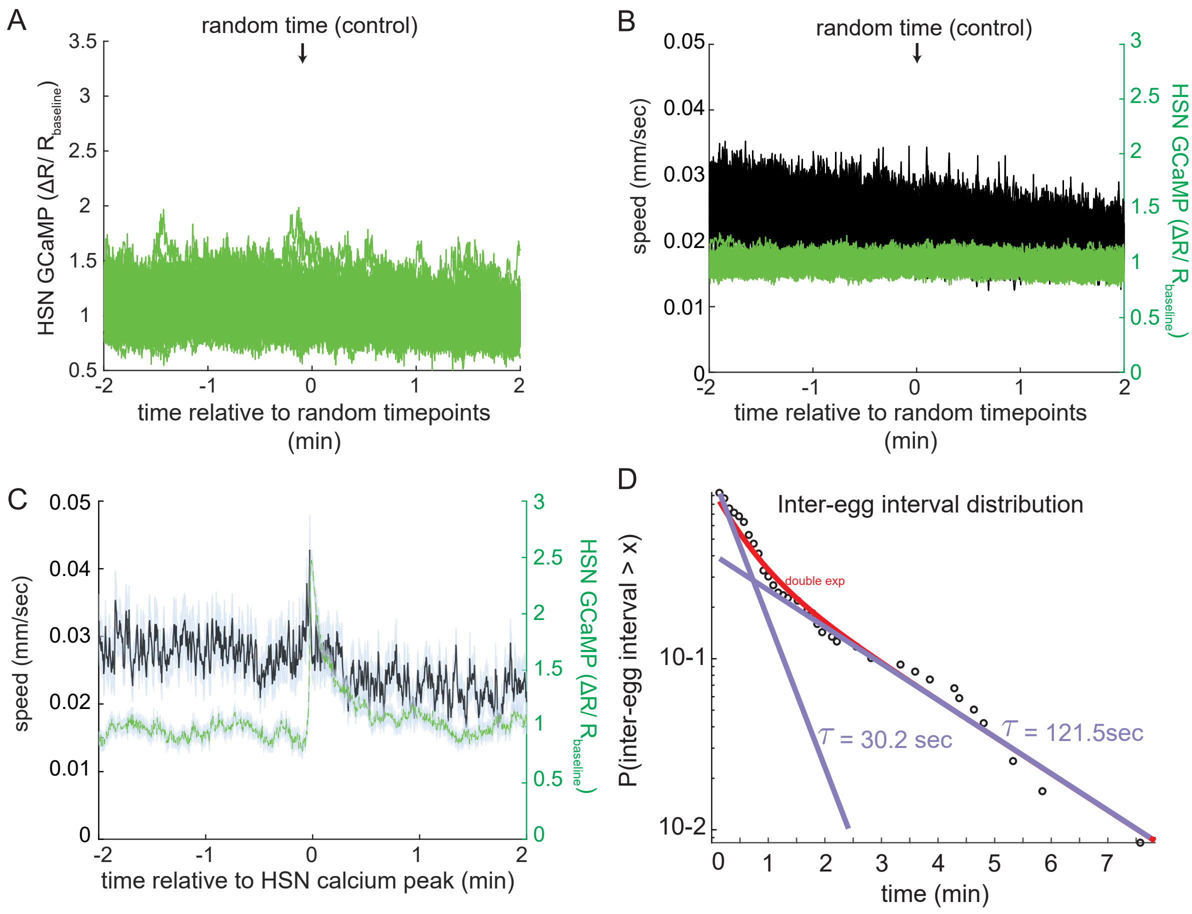


**Figure S2, Related to Figure 2**

(A) Event-triggered averages showing average HSN GCaMP signal surrounding randomly chosen timepoints, as a control for Fig. 1B. Each line is the mean of 16 timepoints (matching the N in Fig. 2B), and this control was run 100 times, resulting in 100 lines.

(B) Event-triggered averages showing average speed (black) and HSN GCaMP (green) surrounding randomly chosen timepoints, as a control for Fig. 2C. Each line is the mean of 104 timepoints (matching the N in Fig. 2C), and this control was run 100 times, resulting in 100 lines.

(C) Event-triggered average showing average animal speed surrounding HSN calcium peaks. This plot only includes HSN calcium peaks that were not accompanied by egg-laying. Note that speeding still occurs during these peaks.

(D) Complementary cumulative distribution function (ccdf) showing distribution of intervals between HSN calcium peaks. This distribution was best fit by a double exponential (red). The slopes of each exponential are shown in blue and the tau values are also displayed. The shorter distribution is characterized by a tau of ~30s, whereas the longer distribution is characterized by a tau of ~2min.

bioRxiv preprint doi: https://doi.org/10.1101/2023.04.02.532814; this version posted April 2, 2023. The copyright holder for this preprint (which was not certified by peer review) is the author/funder, who has granted bioRxiv a license to display the preprint in perpetuity. It is made available under aCC-BY 4.0 International license.

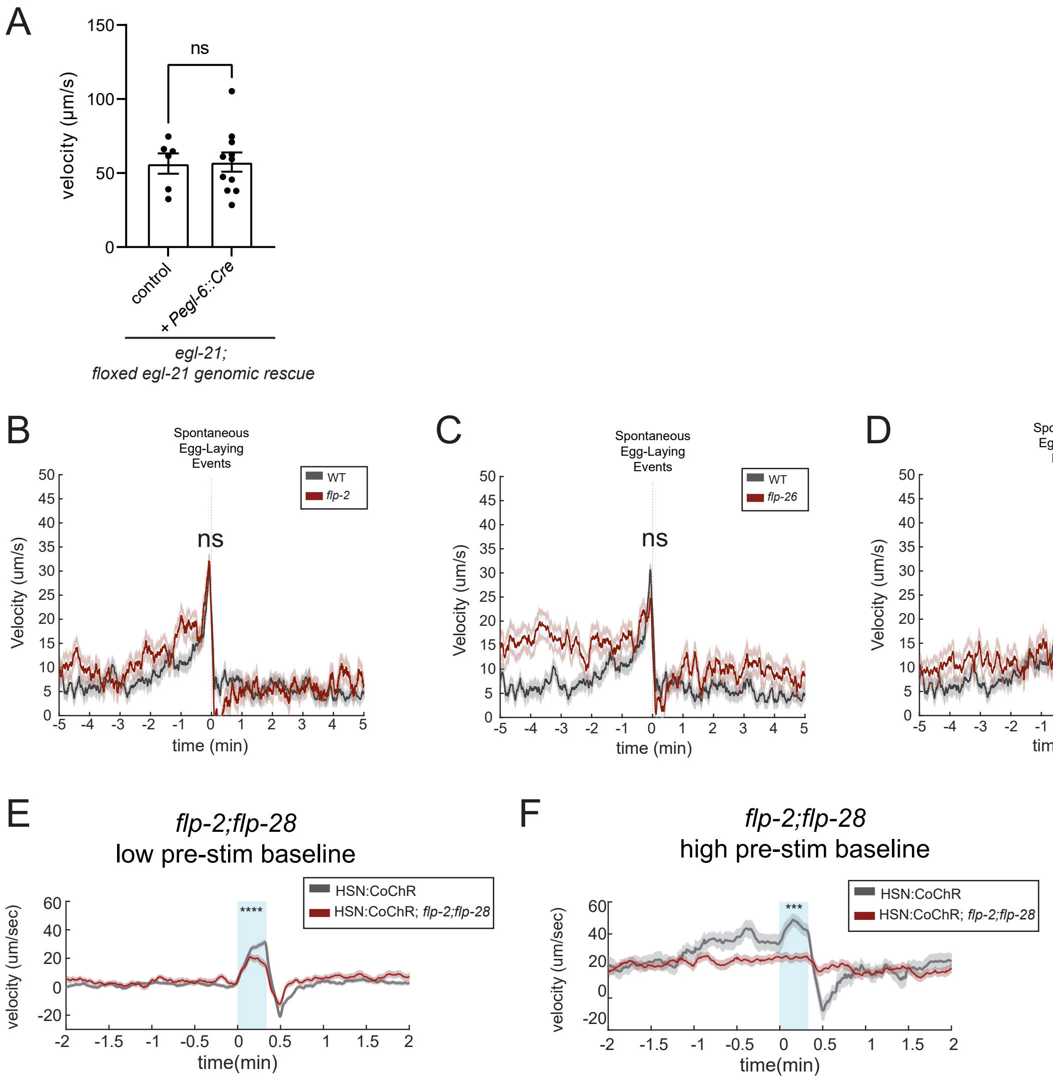


**Figure S3, Related to Figure 3**

(A) Baseline mean velocity of *egl-21; floxed egl-21 genomic rescue* animals without (control) or with *pegl-6::Cre* expression in the absence of food. Dots show individual animals; bars are means and error bars are SEM. N= 6 animals for control and 11 animals for *pegl-6::Cre* expressing animals.

(B) Event-triggered averages for time periods surrounding native egg-laying events in *flp-2(gk1039)* animals. N= 194 egg-laying events across 6 animals.

(C) Event-triggered averages for time periods surrounding native egg-laying events in *flp-26(gk3015)* animals. N= 162 egg-laying events across 6 animals.

(D) Event-triggered averages for time periods surrounding native egg-laying events in *flp-28(flv11)* animals. N= 159 egg-laying events across 6 animals. *$p<0.05$, unpaired t-test.

bioRxiv preprint doi: https://doi.org/10.1101/2023.04.02.532814; this version posted April 2, 2023. The copyright holder for this preprint (which was not certified by peer review) is the author/funder, who has granted bioRxiv a license to display the preprint in perpetuity. It is made available under aCC-BY 4.0 International license.

(E and F) Event-triggered averages showing average changes in velocity in *flp-2(flv15);flp-28(flv11)* animals in response to HSN::CoChR activation via light illumination. Gray provides data from WT animals as a control and reference. Lines show means and error shading shows SEM. (D): Data with animals dwelling at low velocity (<20µm/sec) before light illumination. N =249 stimulation events for WT and 129 stimulation events for *flp-2(flv15);flp-28(flv11)* animals. (E): Data with animals having higher velocity baseline (>=20µm/sec) before light illumination. N =21 stimulation events for WT and 46 stimulation events for *flp-2(flv15);flp-28(flv11)* animals. ****p<0.0001, unpaired t-test.

bioRxiv preprint doi: https://doi.org/10.1101/2023.04.02.532814; this version posted April 2, 2023. The copyright holder for this preprint (which was not certified by peer review) is the author/funder, who has granted bioRxiv a license to display the preprint in perpetuity. It is made available under aCC-BY 4.0 International license.

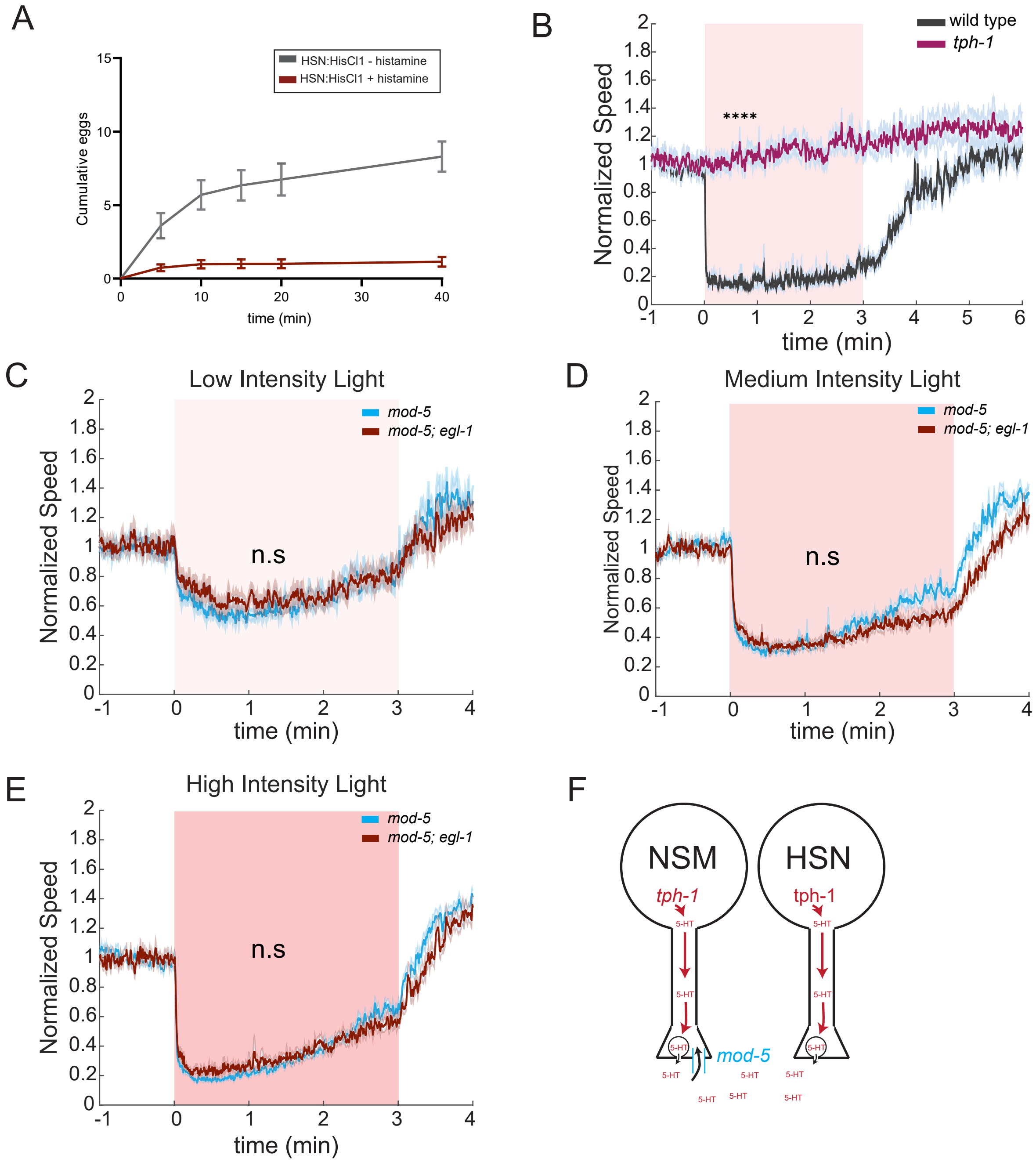


**Figure S4, Related to Figure 4**

(A) Egg-laying of HSN::HisCl animals either exposed to histamine or not. Animals for this experiment were transferred to +his or -his plates immediately before this assay (i.e. at t=0min), and eggs laid were counted at different time points, up to 40min after transfer. Note that egg-laying is reduced even at the first time point, indicating the HSN::HisCl inhibits egg-laying

bioRxiv preprint doi: https://doi.org/10.1101/2023.04.02.532814; this version posted April 2, 2023. The copyright holder for this preprint (which was not certified by peer review) is the author/funder, who has granted bioRxiv a license to display the preprint in perpetuity. It is made available under aCC-BY 4.0 International license.

within minutes of first exposure to histamine. N=35 plates for HSN::HisCl with histamine and 20 plates for no histamine control group, with 3 animals on each plates.

(B) Event-triggered averages depicting the average change in animal speed upon NSM::Chrimson stimulation with red light illumination in wild-type and *tph-1(mg280)* animals. Animals were starved for 3 hours before the assays. Lines show means; and error shading shows SEM. N= 77-390 animals. ***$p<0.0001$, unpaired t-test.

(C to E) Event-triggered averages depicting the average change in animal speed upon NSM::Chrimson stimulation with different light intensity in *mod-5(n822)* and *mod-5(n822);egl-1(n487gf)* animals, with panel C being the lowest intensity and panel E the highest. N= 51-248 animals.

(F) Cartoon illustrating serotonin release and re-uptake by NSM and HSN neurons.
